# Sarcoidosis as an unusual cause of unexplained pancytopenia

**DOI:** 10.1002/jha2.650

**Published:** 2023-01-31

**Authors:** Caitlin Raymond, Sri Bharathi Kavuri, Faisal Rawas, Jayati Mallick, Juan Olano, Kirill A. Lyapichev

**Affiliations:** ^1^ Department of Pathology The University of Texas Medical Branch Galveston Texas USA

**Keywords:** bone marrow failure, bone marrow pathology, chronic disease, corticosteroids, immunohaematology, pathology

1

Sarcoidosis is a systemic inflammatory disease of unknown etiology characterized by noncaseating granulomata, most commonly in lung, skin, and lymph nodes. However, sarcoidosis can affect any tissue, including the bone marrow, although this finding is very rare (0.3%–2.2% of bone marrow biopsies) [1].

A 35‐year‐old male presented with hematochezia and hemoptysis. He was consequently found to have pancytopenia with diffuse lymphadenopathy and marked splenomegaly. The patient's complete blood count (CBC) was notable for severe panleukopenia with total white blood cell count of 1.45 × 10^^3^, normocytic, normochromic anemia with hemoglobin of 9.5 g/dl, and thrombocytopenia to 72 × 10^^3^. Blood cultures were negative for pathogenic organisms. Bone marrow biopsy was performed.

Histological findings revealed noncaseating granulomata with moderate myelofibrosis involving approximately a third of the bone marrow (Figure 1A). Special stains for reticulin and trichrome showed moderate myelofibrosis (MF‐2) (Figure 1B–C). CD68 highlights dense infiltration of histiocytes into the bone marrow and granulomata (Figure 1D). CD3, CD20, and CD56 highlight scattered infiltrating T‐ and B‐lymphocytes, and natural killer (NK)‐cells. Grocott's methenamine silver (GMS) and acid‐fast bacteria (AFB)/Fite stains were negative for microorganisms. Next generation sequencing was performed and was negative for mutations in JAK2, MPL, and CALR.

**FIGURE 1:**
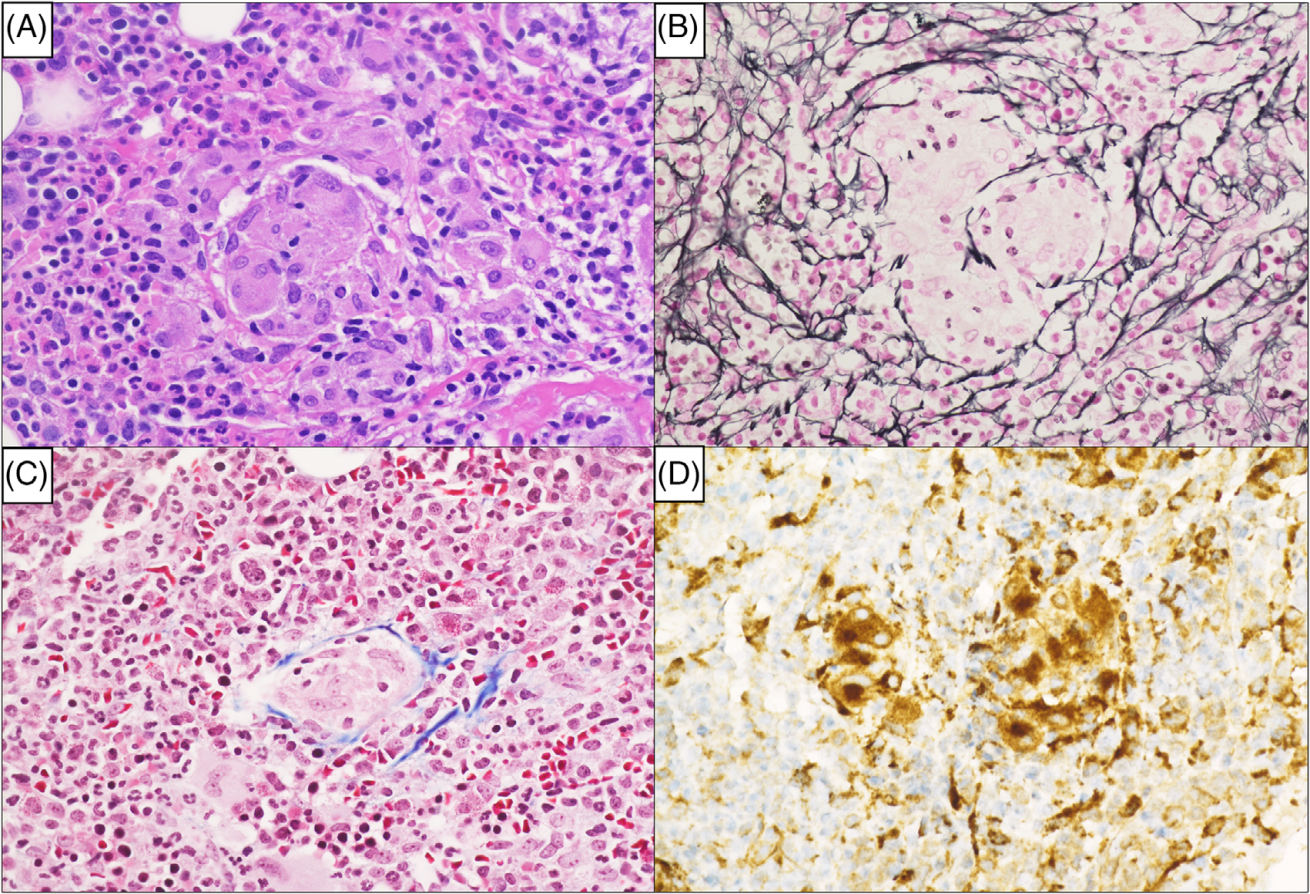
Noncaseating granulomas in the bone marrow of a patient with preexisting diagnosis of sarcoidosis. (A) H&E staining at 400× of a single noncaseating granuloma. (B) Retiulin staining at 400x demonstrating moderate fibrosis (MF‐2) encasing a granuloma. (C) Trichrome staining at 400× demonstrating moderate (MF‐2) fibrosis around a granuloma. (D) CD68 staining highlights histiocytes infiltrating the bone marrow and granulomata.

After extensive clinical history review, we discovered an extant diagnosis of sarcoidosis for which the patient was unable to access treatment. Based on this prior clinical history and our histological findings, the diagnosis of sarcoidosis involving bone marrow was established [2].

Granulomas are a nonspecific finding often seen in infections, particularly tuberculosis and fungal. In this case, all infectious work up—including GMS and AFB stains—was negative. Sarcoidosis classically involves lungs, skin, and lymph nodes. Involvement of the bone marrow is a rare finding, estimated to be present in only 10%–17% of all sarcoidosis cases [3].

This case highlights the progression of untreated sarcoidosis, ultimately leading to infiltration of the bone marrow. The resulting suppression of hematopoiesis is the likely cause of the patient's pancytopenia [4].

Following identification of sarcoidosis involvement of bone marrow, the patient was treated with high dose methylprednisolone. Following two weeks of treatment, CBC numbers improved significantly with white blood cells of 7.75 × 10^3^, hemoglobin of 10.1 g/dl, and platelets of 145 × 10^3^.

## AUTHOR CONTRIBUTIONS

Caitlin Raymond wrote the manuscript, assisted in taking original images, and produced the figure. Sir Kavuri assisted in taking original images, and provided valuable contributions on the manuscript. Faisal Rawas produced the slides from which images were taken. Jayati Mallick and Juan Olano provided valuable contributions to the manuscript. Kirill Lyapichev assisted in taking original images, and provided valuable comments on the manuscript.

## FUNDING INFORMATION

There were no sources of funding for this manuscript.

## CONFLICT OF INTEREST

The authors have no conflict of interest to disclose.

## ETHICS STATEMENT

The information presented in this manuscript is deidentified, and there is minimal risk to the patient's privacy or confidentiality. IRB approval was not required by our institution for preparation of this manuscript. Patient consent was not obtained. No material from other sources is included in this manuscript.

## Data Availability

Data from this manuscript will be shared upon request
